# Editorial: Wearable and mobile data analysis methodologies for personalized medicine

**DOI:** 10.3389/fdgth.2023.1271659

**Published:** 2023-09-20

**Authors:** Ivan Miguel Pires, Ciprian Dobre, Eftim Zdravevski, Nuno M. Garcia

**Affiliations:** ^1^ Instituto de Telecomunicações, Covilhã, Portugal; ^2^ Escola Superior de Tecnologia e Gestão de Águeda, Universidade de Aveiro, Águeda, Portugal; ^3^ Department of Computer Science, Polytechnic University of Bucharest, Bucharest, Romania; ^4^ Faculty of Computer Science and Engineering, Saints Cyril and Methodius University, Skopje, North Macedonia; ^5^ Faculty of Sciences, University of Lisbon, Lisbon, Portugal

**Keywords:** personalized medicine, precision medicine, wearable devices, health tracking, methodology, big data, data fusion

**Editorial on the Research Topic** Wearable and mobile data analysis methodologies for personalized medicine

The Frontiers in Digital Health Research Topic *Wearable and Mobile Data Analysis Methodologies for Personalized Medicine* aimed to receive contributions related to the multidisciplinary field of personalized and precision medicine, which encompasses physics, statistics, telemedicine, biomedical engineering, digital signal processing, artificial intelligence, system engineering, and health privacy and security.

Information and communication technologies have changed the landscape of many knowledge and societal areas, and medicine included. Bringing technology to the end users (or patients), a larger share of users can engage in personalized and precision therapies. Numerous and diverse pathologies can be monitored remotely, and as a consequence, better monitoring of health-related information may not only empower people but also hold the promise of aiding in the early detection of diseases.

Devices like smartphones, smartwatches, and others are equipped with sensors that may gather various data and facilitate interactions between the communities of patients, care givers and healthcare professionals. These devices can be used in a vision of a health care architecture where the wearable and home devices are a pilar that supports a more preventive, predictive, precise, participative, and personalized medicine, contributing to better diagnosis and therapeutics. Yet, for this to happen, the research community must investigate how cutting-edge technology, such as mobile and wearable devices, artificial intelligence, big data, data fusion, and data imputation, might be used to make true this vision of a new type of health care approach.

This Research Topic has four articles that were accepted after the meticulous and rigorous review process, and those are summarized in the remainder of this editorial.

One of the critical areas in personalized medicine can be related to psychological issues, and the authors of (Jungnickel et al.) recommended examining a framework-based strategy for mobile apps for the execution of psychological testing based on Apple's ResearchKit. It was discovered that rather than the particulars of the selected platform or input method, the validity of the gathered measurements depended on the characteristics of the selected test.

In (Scheid et al.), the researchers created a method to assess the viability of measuring motor function in Huntington's disease patients using wearable sensors and machine learning algorithms. The developed method makes it possible to access the various scores, enabling the use of biosensors in the future for an objective assessment of Huntington's disease in a clinic or remotely. This method may also guide future research on using this technology as a potential endpoint in clinical trials.

The authors of (Carboni et al.) began with a state-of-the-art privacy presentation for European Active Healthy Ageing/Active Healthy Ageing projects, focusing on those that used audio and video processing. Then, they put forth a methodology created as part of the European project PlatfromUptake.eu to identify stakeholder groups and application dimensions (technical, contextual, and business), define their properties, and illustrate how privacy restrictions affect them. We then used the results of this study to create a Strengths, Weaknesses, Opportunities, and Threats analysis, in which we try to pinpoint the critical elements associated with the choice and participation of the right stakeholders for a project's success.

Finally, the authors of (Nemer et al.) concentrated on the identification of digital reproductive, maternal, newborn, and child health (RMNCH) initiatives in Palestine and Jordan and mapping pertinent data from identified initiatives. The information was gathered from various resources, including pertinent documents already available and direct communications with stakeholders. The study demonstrates how digital health integrates into the Palestinian and Jordanian healthcare systems. However, there needs to be a clear regulatory standard in place, particularly regarding the privacy and security of personal data and how it is managed.

The editors of this Research Topic—Ivan Miguel Pires of Instituto de Telecomunicações, Covilhã, Portugal, and Polytechnic Institute of Santarém, Santarém, Portugal; Ciprian Dobre of Polytechnic University of Bucharest, Bucharest, Romania; Eftim Zdravevski of Saints Cyril and Methodius University, Skopje, Macedonia; and Nuno M. Garcia of the Faculty of Sciences, University of Lisbon, Lisbon, Portugal, and Instituto de Telecomunicações, Covilhã, Portugal—hope that the published papers could serve as a starting point for new research in personalized medicine methods.

